# Supplementary material for: Differential nuclear import sets the timing of protein access to the embryonic genome
Source: Nat Commun. 2022 Oct 6;13:5887. doi: 10.1038/s41467-022-33429-z (PMC9537182; doi:10.1038/s41467-022-33429-z)
Supplement: Supplementary file 1 — Supplementary Information [file 41467_2022_33429_MOESM1_ESM.pdf]

Supplementary Figures

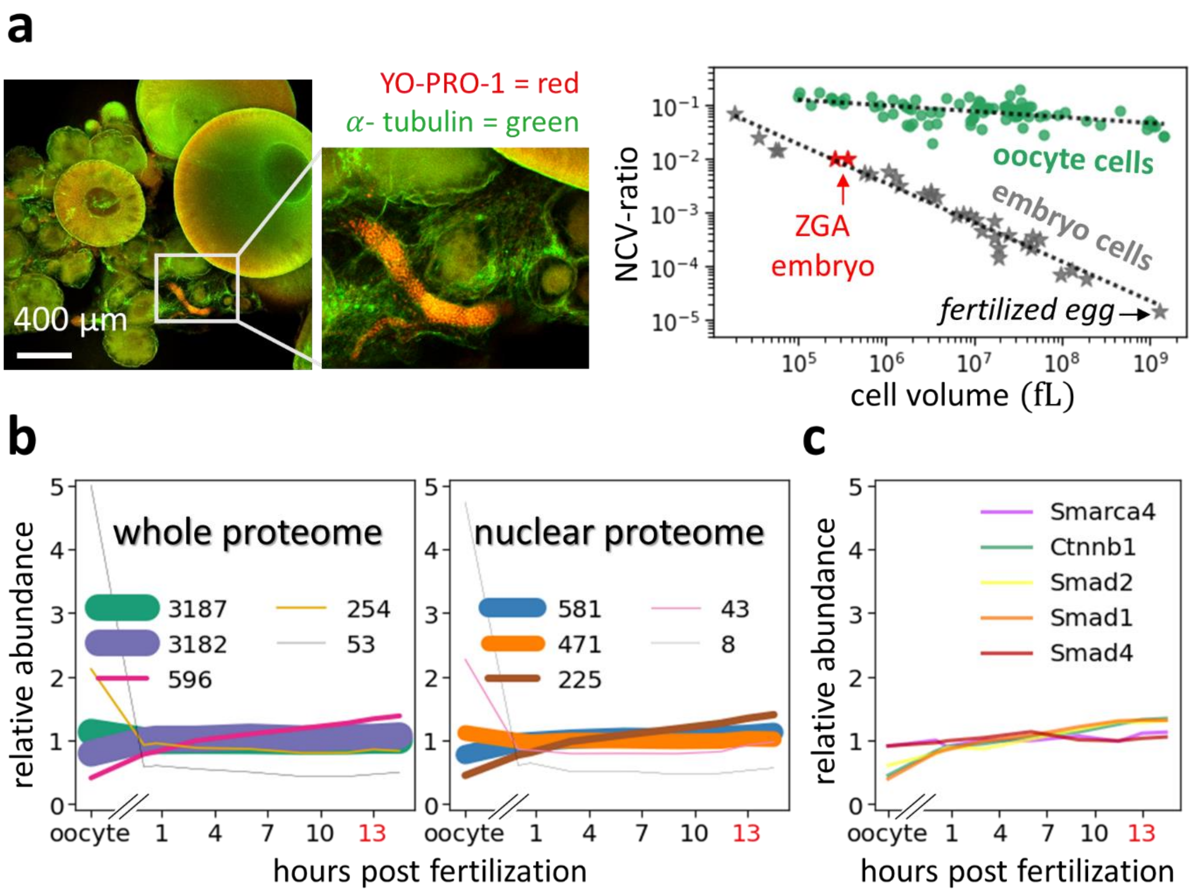

Supplementary Figure 1: Changes in nucleocytoplasmic volume (NCV) ratio for cells in the frog ovary and early development.

a, Left: Immunofluorescence (α-tubulin and Yo-Pro-1) of a frog ovary shows cells varying by 3-orders of magnitude in diameter (from ~10µm to ~1200µm). A zoomed-in section of the white framed box is shown to the right. The represented images were independently repeated multiple times for reproducibility and observed with similar results.

Right: Quantification of the NCV-ratio versus cell volume for maturing oocytes and early developing embryos. Compared to the rapidly changing embryos, the oocyte NCV-ratios remain approximately constant (decreasing by 2.3-fold as the cell volume increases ~500-fold (from ~2nL to ~1µL)) throughout oogenesis. The slight decrease might come from some cytoplasmic volume being excluded by the forming yolk platelets[1,2]. In contrast, the NCV-ratios increase by ~34,000-fold in developing embryos from a ~1µL fertilized egg to ~30pL cells at 46 hours post-fertilization (16°C). (Supplementary Data 5).

b, k-means clustering (k = 5) of changes in relative protein abundance of the entire proteome (left) and nuclear proteins (right) from the time series of the oocyte to ZGA embryo measured by multiplexed proteomics. The line thickness scales with the number of proteins within a cluster. While the NCV-ratio drastically increases from the fertilized egg to the ZGA, protein levels change little.

c, The detected transcription factors reported being involved in the gene regulatory network of the mesendoderm formation do not change their expression levels between fertilization to the ZGA[3].

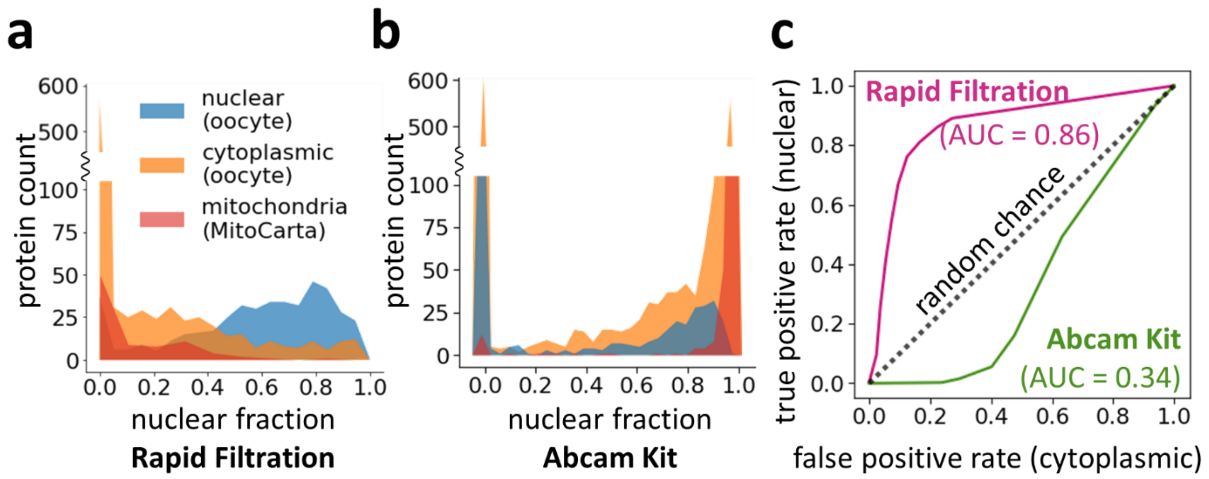

Supplementary Figure 2: Rapid nuclear filtration outperforms nuclear isolation method based on differential sedimentation in quantifying the nucleocytoplasmic (NC) partitioning in early frog embryos.

a, The newly developed rapid filtration method can enrich nuclear protein signals from the rest of the embryonic cell. Shown is the histogram of nuclear fraction, quantified with multiplexed proteomics, after the fractionation of ZGA embryos using the nuclear filtration method.

b, Nuclear extraction based on differential sedimentation poorly separates nuclear proteins from the proteins of other organelles. Shown is the quantification as in (**a**) using a commercial nuclear extraction kit (Abcam).

c, Receiver operating characteristics (ROC) for the measured nuclear fractions, comparing filtration and sedimentation-based nuclear enrichment methods. We use proteins that were measured nuclear in the oocyte as true positives and proteins that were measured cytoplasmic in the oocytes as false positives[4]. Measuring NC partitioning via rapid filtration (pink) appears superior to sedimentation (green) in early frog embryos.

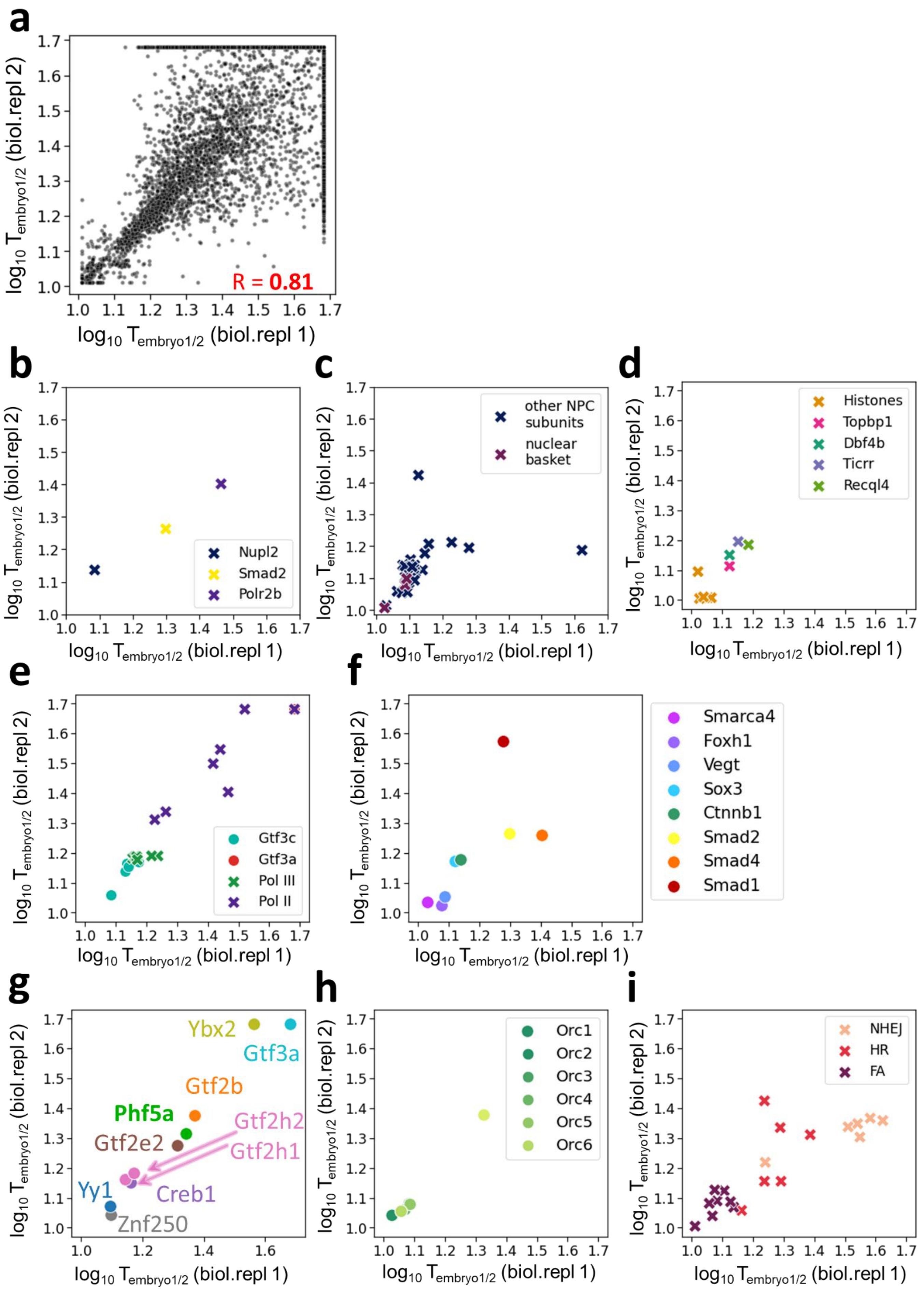
a
R = 0.81
log10 Tembryo1/2 (biol.repl 1)
log10 Tembryo1/2 (biol.repl 2)
b
Nupl2
Smad2
Polr2b
c
other NPC subunits
nuclear basket
d
Histones
Topbp1
Dbf4b
Ticrr
Recql4
e
Gtf3c
Gtf3a
Pol III
Pol II
f
Smarca4
Foxh1
Vegt
Sox3
Ctnnb1
Smad2
Smad4
Smad1
g
Ybx2
Gtf3a
Gtf2b
Phf5a
Gtf2h2
Gtf2e2
Gtf2h1
Yy1
Creb1
Znf250
h
Orc1
Orc2
Orc3
Orc4
Orc5
Orc6
i
NHEJ
HR
FA

Supplementary Figure 3: The level of reproducibility of the embryonic nuclear proteome experiments on the proteome-wide scale and on individual groups of protein discussed throughout the manuscript.

a, Scatter plot of two biological replicates to determine $T_{\mathrm{embryo}1/2}$ of nuclear entry into embryonic nuclei. We observe a Pearson correlation of 0.81 (two-tailed p-value < 1e-325) with lower reproducibility at late $T_{\mathrm{embryo}1/2}$.

b-i, Correlation plots for highlighted proteins discussed in the manuscript are separated into different panels, corresponding to the associated figures in the main text. Below are the pairs of the presented panel and the corresponding figure in the manuscript: panel (**b**) – Fig. 2b, panel (**c**) – Fig. 2e & Supplementary Fig. 3b, panel (**d**) – Fig. 3a, panel (**e**) – Fig. 3b & Supplementary Fig. 4, panel (**f**) - Fig. 3c, panel (**g**) – Fig. 5c, panel (**h**) – Supplementary Fig. 4a, panel (**i**) – Fig. 2d.

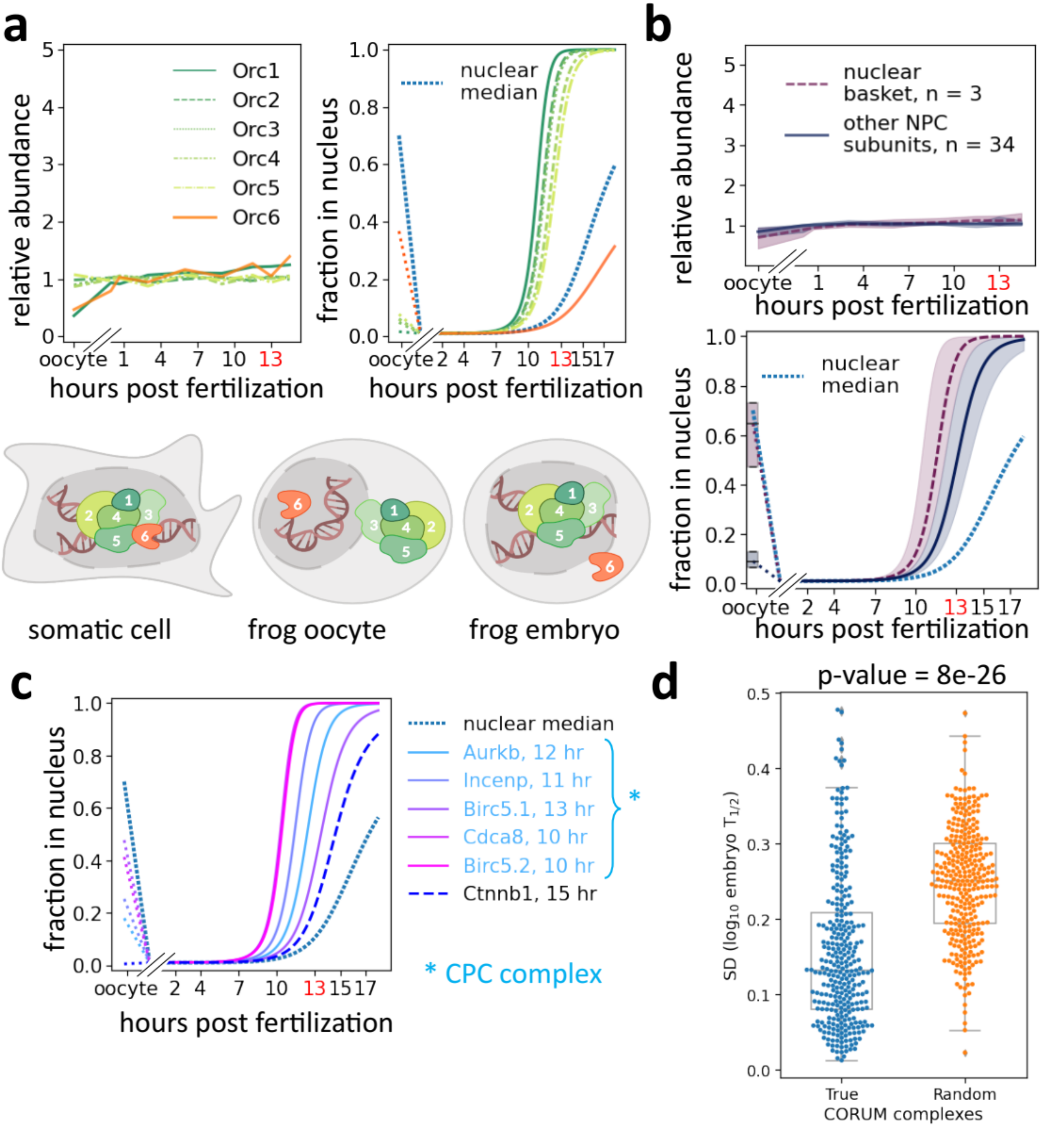

a
relative abundance
Orc1
Orc2
Orc3
Orc4
Orc5
Orc6
oocyte
1
4
7
10
13
hours post fertilization
fraction in nucleus
nuclear median
oocyte
2
4
7
10
13
15
17
hours post fertilization
somatic cell
frog oocyte
frog embryo
b
relative abundance
nuclear basket, n = 3
other NPC subunits, n = 34
hours post fertilization
fraction in nucleus
nuclear median
hours post fertilization
c
fraction in nucleus
nuclear median
Aurkb, 12 hr
Incenp, 11 hr
Birc5.1, 13 hr
Cdca8, 10 hr
Birc5.2, 10 hr
Ctnnb1, 15 hr
* CPC complex
hours post fertilization
d
p-value = 8e-26
SD (log10 embryo T1/2)
True
Random
CORUM complexes

Supplementary Figure 4: Quantifying protein NC partitioning reveals sequential nuclear entry in early embryos.
a-d, embryos developed at 16°C. Solid lines indicate median nuclear titration patterns and shaded areas 50% spread.
a, Core subunits of the origin of replication complex (ORC) enter the nucleus at different times.
Left: Relative protein abundances of ORC subunits stay approximately constant from fertilization to ZGA.
Right: Orc6 enters embryonic nuclei later than other components, consistent with previous observations of cytoplasmic function in *Drosophila* and mammals[5]. While Orc1-6 localize in somatic nuclei, Orc1-5 are cytoplasmic and Orc6 is nuclear in the oocyte[4,6]. In early embryos, Orc1-5 rapidly enter the nuclei, presumably to accommodate for function in *Xenopus* embryos' DNA replication process[7].
Bottom: Cartoon model summarizing NC partitioning of ORC core subunits in the oocyte, embryo, and somatic cells.
b, Nuclear basket proteins show different NC partitioning in early development than other nuclear pore complex (NPC) proteins.
Top: Yet, nuclear basket proteins demonstrate constant expression levels during early development.
Bottom: The nuclear basket is mostly nucleus-localized, contrasting NPC proteins being generally equidistributed between nucleus and cytoplasm in the oocyte. Nuclear basket proteins titrate into the nucleus slightly faster than other subunits. Previous observations indicate nuclear baskets are not part of the annulate lamellae but interact with actin filamentous networks connecting NPC to chromatin in oocyte nuclei[8].
c, The CPC complex was equidistributed between the nucleus and cytoplasm in the oocyte. However, CPC components is sequestrated into the embryonic nuclei. The transcription factor β-catenin (Ctnnb1) is cytoplasmic in the oocyte but is among early nuclear-imported proteins[9,10].
d, Protein complex subunits co-import into nuclei. The standard deviation of nuclear entry time of each complex measured in the embryonic assay (blue) versus those of complexes with shuffled subunits (orange)[11] (Wilcoxon-rank two-sided test with N=311 biological independent complexes p-value ~8e-26). Box plots of the range from Q1 to Q3 and 1.5* IQR whiskers are shown.

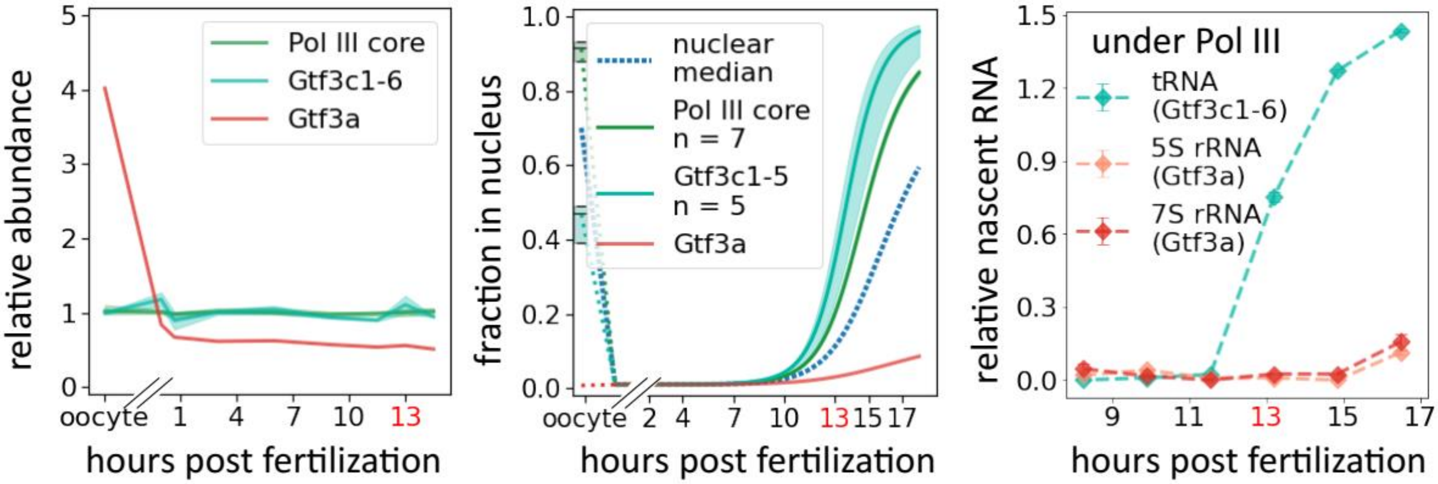

Supplementary Figure 5: Nuclear entry of Pol III and its transcription factors during early development.

Embryos developed at 16°C. For complexes with multiple subunits, we represent the median nuclear titration pattern by a solid line and a 50% spread by shaded area with the corresponding color. Source data are provided as Supplementary Data 1,2.

Left: Relative protein abundance of Pol III and its associated transcription factors, Gtf3a and Gtf3c1-5, stay approximately constant from fertilization to the ZGA.

Middle: Although the core Pol III titrates in the nucleus early, the nuclear entry times of its associated transcription factors vary. In our proteomics analysis, Gtf3c1-5 titrate in the nucleus before Gtf3a. Under Pol III, the transcription factors Gtf3c1-5 (and Pol III) are upstream of tRNAs, while Gtf3a (and Pol III) are upstream of 5S rRNA and 7S rRNA.

Right: Quantification of the newly transcribed RNA indicates that tRNA is transcribed much earlier than 5S rRNA and 7S rRNA, which corresponds to the nuclear entry order of their upstream transcription factors. Quantification is from Newport and Kirschner's RNA gel of newly synthesized transcripts[12].

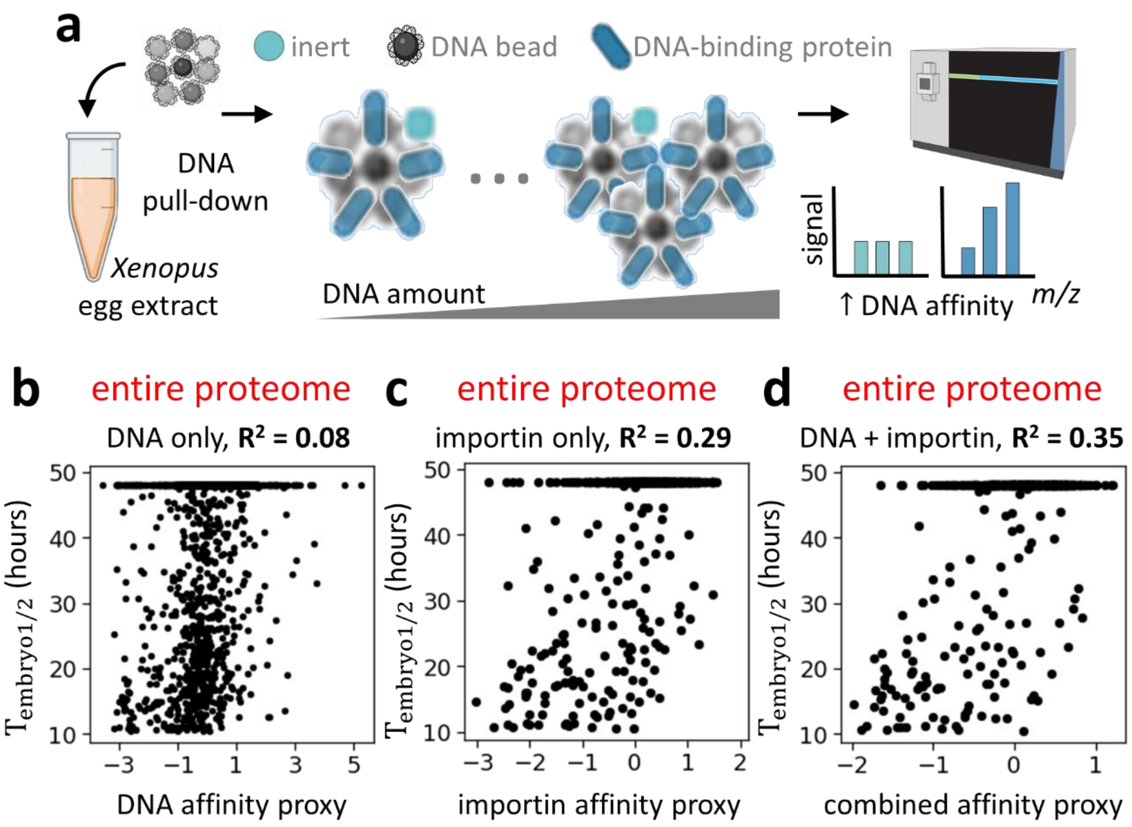

Supplementary Figure 6: Quantification of proteome-wide affinities to plasmid DNA and their correlation with nuclear entry times in early embryos.

a, Quantification of proteome-wide protein affinity to DNA. We exposed *Xenopus* egg lysate to magnetic beads that were covered with plasmid DNA. The pull-down was collected and subjected to MS quantification of the relative protein signal. The experiment was repeated with different DNA to lysate ratios. The DNA affinities at different ratios were projected on one dimension of the canonical coordinate space with cross-validation to produce a DNA affinity proxy for each identified protein[13].

b, Scatter plot of the projected DNA affinity proxy versus $T_{\mathrm{embryo1/2}}$. The correlation suggests that plasmid DNA affinity explains 8% of the nuclear import variance in the embryos. For technical reasons, we could not perform these assays with frog DNA. Therefore, this assay does not capture protein affinity to frog-specific DNA sequences.

c, Scatter plot of the importin affinity proxy versus $T_{\mathrm{embryo1/2}}$ for all quantified proteins suggests that importin affinities can explain 29% of the of the nuclear import variance in the embryos.

d, The combined DNA affinities and importin affinities projected onto a single dimension result in an improved correlation with nuclear entry time, suggesting that together DNA and importin affinities can explain >35% of the variance of observed timing of nuclear entry in early embryos. Shown is the scatter plot between $T_{\mathrm{embryo1/2}}$ and the projected affinity proxy.

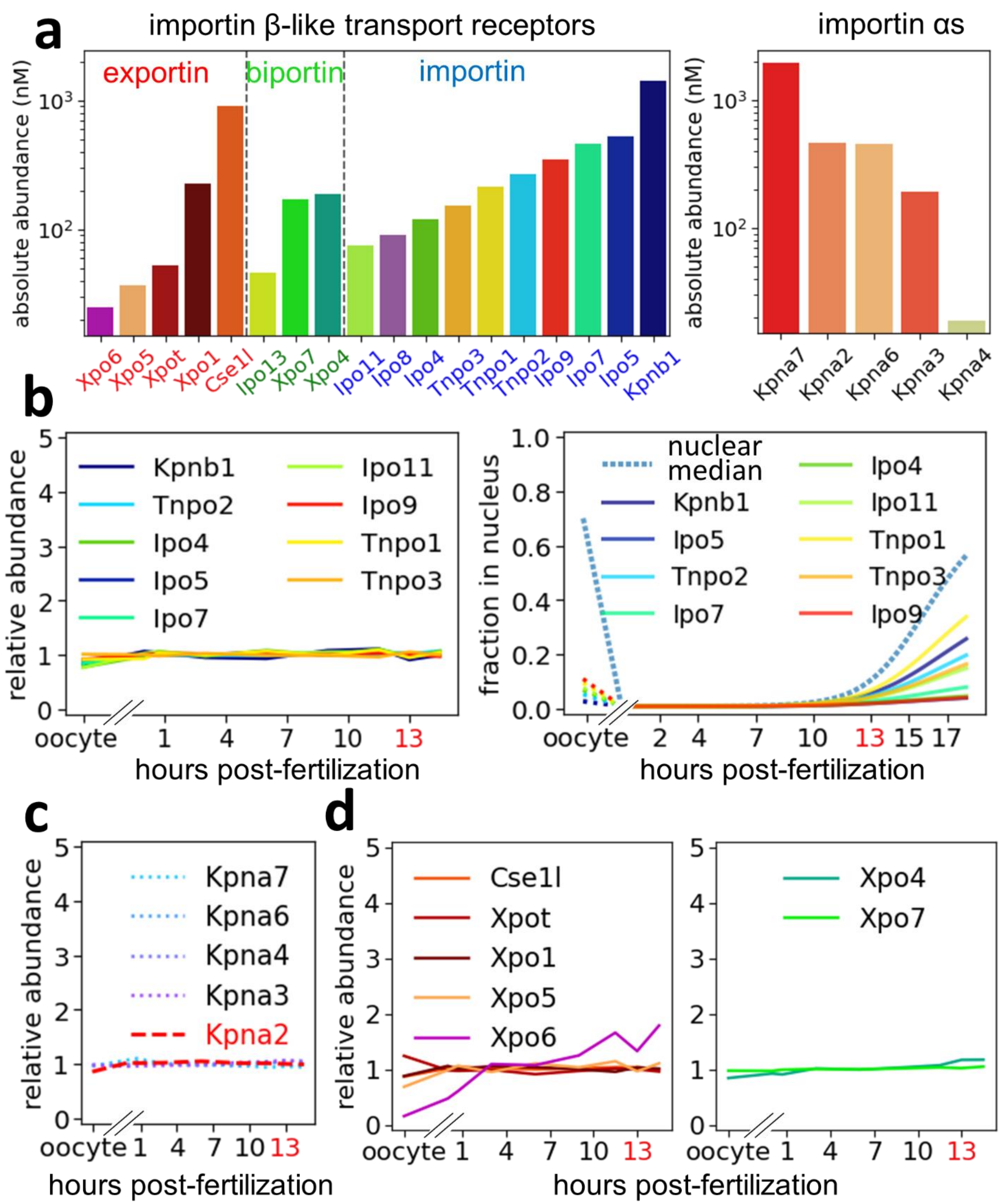

Supplementary Figure 7. The expression levels and subcellular localization of nuclear transport receptors observed in the frog embryos and their dynamic changes in early development.

Source data are provided as Supplementary Data 1,2,4.

a, The absolute abundance of transport receptors in the frog egg. Left: importin β family, right importin αs (Supplementary Data 4[4]).

b, Relative protein abundance (left) and the subcellular localization (right) of the importin β family importins as a function of developmental progression. We observed that the expression levels of

importins stay constant throughout development and that the proteins locate preferentially in the cytoplasm throughout early development. The preferential cytoplasmic partition of importins was similarly observed in the frog oocyte[4].

c, Relative protein abundances of importin α's in early frog embryos show that their levels remain approximately constant.

d, Relative protein abundance changes of exportins and biportins as a function of developmental progression. Most exportins and biportins remain approximately constant. The exception is exportin 6 (Xpo6), which has actin as the primary substrate. Xpo6 is absent in Xenopus oocytes, which results in nuclear actin localization that supports the physical integrity of the large oocyte nucleus[14,15]. Upon fertilization, Xpo6 expression level increases, and actin is excluded from embryonic nuclei.

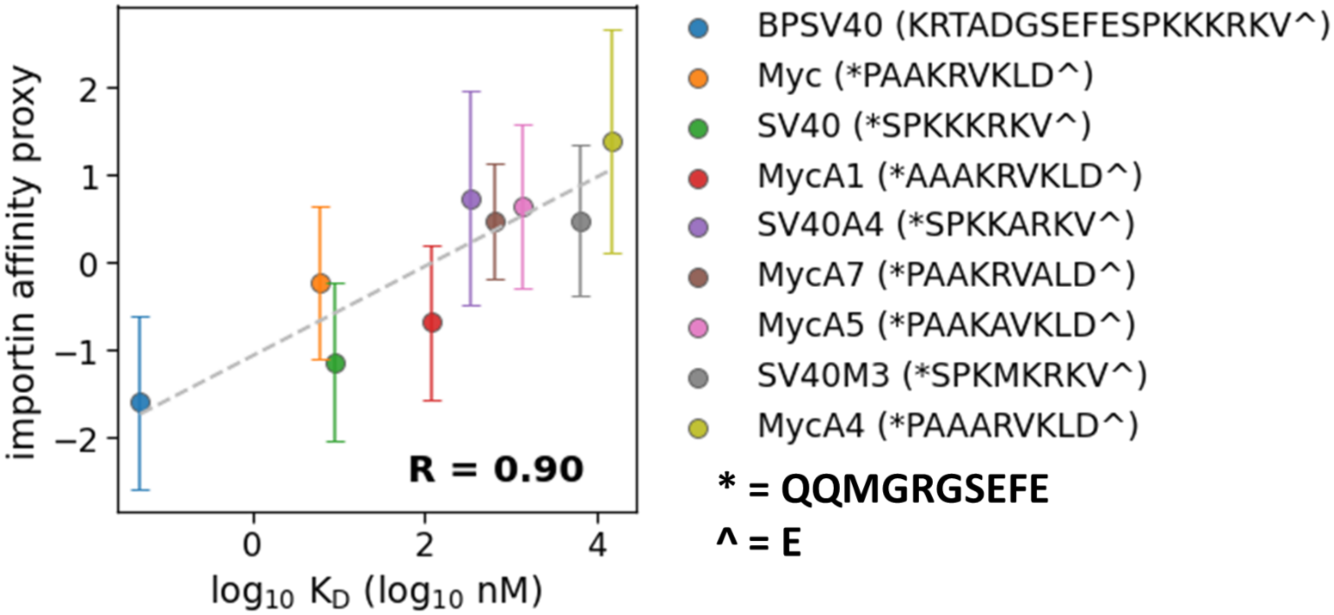

Supplementary Figure 8: Validation of importin affinity assay (Figure 4) using NLS peptides with orthogonally measured K_Ds[16]. We defined the importin affinity proxy as the free peptide concentration difference between a condition with RanQ69L and a condition without RanQ69L (importin affinity proxy = [free protein+RanQ69L] – [free protein–RanQ69L]). We observed a correlation of 0.90 (R^2 = 0.80, two-tailed p-value = 0.001) between our measured importin affinity proxy with the log K_Ds from Hodel et. al.[16] The line is a linear fit. Solid dots are the means of importin affinity proxy and error bars indicate standard error (calculated from a set of biological independent replicates, n = 3 controls and 5 RanQ69L added conditions, resulting in 15 biological independent measurements of importin affinity proxy).

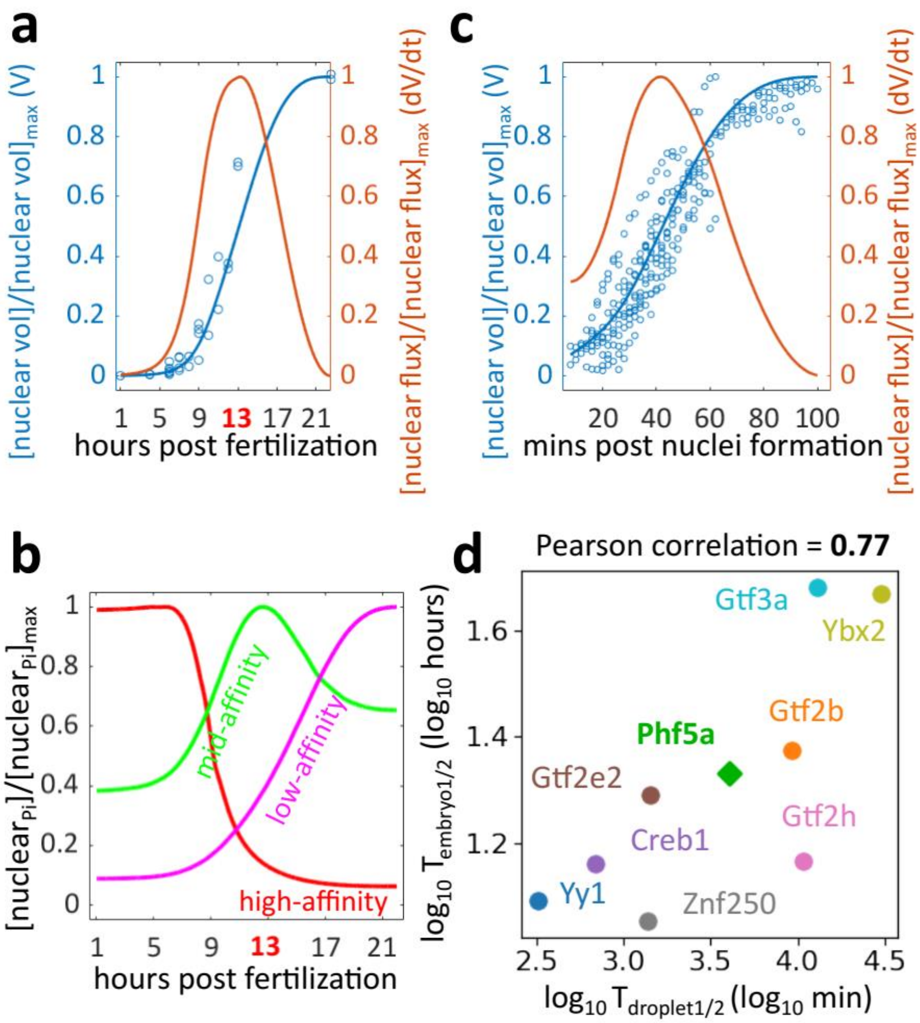

Supplementary Figure 9: Changes of total nuclear volume in embryos and droplet assay; predictions of nuclear concentration for proteins with varying importin affinity in embryos; Scatter plot of nuclear import timing cell-free assay and embryos.

a, Total nuclear volume, and nuclear protein flux as functions of time in developing *X. laevis* embryos. Each dot indicates measurements of total nuclear volume based on immunofluorescence from one embryo. A spline fit (blue curve) provides the functional form of the changes in nuclear volume over time. The time derivative of nuclear volume expansion is the nuclear flux due to nuclear import (orange curve). The maximum volume during this time normalizes the nuclear volume (y-axis left), and the nuclear flux is normalized by the maximum flux (y-axis right). The decline in the total nuclear import rate might be due to the previously reported sequestration of importin α to the cellular membrane[17,18].

b, Our model predicts the changes in nuclear concentration over increasing embryonic nuclear volume for three proteins with different affinities to importin. The three proteins sequentially reach their maximal nuclear concentration from the highest affinity protein to the lowest affinity protein. After the maximum is reached, a further nuclear volume increase leads to a decline in nuclear concentration.

c, Nuclear volume and nuclear flux as functions of time in oil encapsulated cytoplasmic droplets. Similar to (**a**), the nuclear volume per cell-free droplet is shown to increase with time in the raw imaging data (blue dots) and is fit by a spline function (blue curve). The function's time derivative is the nuclear flux over time (orange curve). $T = 0$ is when the cytoplasm is taken off ice, and nuclear formation is initiated.

d, Comparing the time measurement of nuclear import in ($T_{\text{droplet1/2}}$) from the cell-free assay and the log import time ($T_{\text{embryo1/2}}$) for the nine TFs show strong agreement (Pearson correlation of 0.77, two-tailed p-value = 0.01).

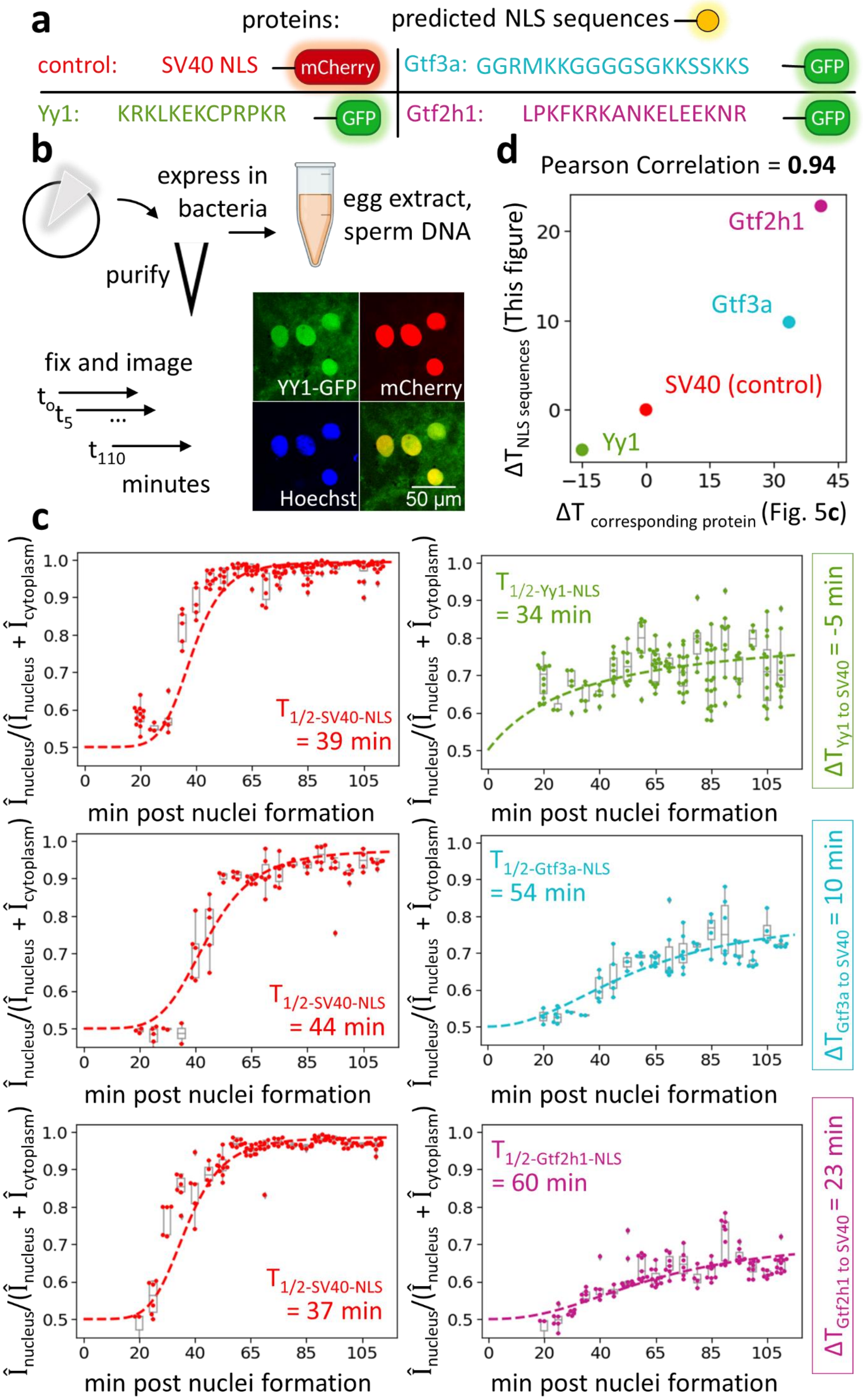

a
proteins:
predicted NLS sequences
control:
SV40 NLS
mCherry
Gtf3a:
GGRMKKGGGGSGKKSSKKS
GFP
Yy1:
KRKLKEKCPRPKR
GFP
Gtf2h1:
LPKFKRKANKELEEKNR
GFP
b
express in bacteria
purify
egg extract, sperm DNA
fix and image
minutes
YY1-GFP
mCherry
Hoechst
50 μm
d
Pearson Correlation = 0.94
Gtf2h1
Gtf3a
SV40 (control)
Yy1
ΔT NLS sequences (This figure)
ΔT corresponding protein (Fig. 5c)
c
T 1/2-SV40-NLS = 39 min
T 1/2-Yy1-NLS = 34 min
ΔT Yy1 to SV40 = -5 min
T 1/2-SV40-NLS = 44 min
T 1/2-Gtf3a-NLS = 54 min
ΔT Gtf3a to SV40 = 10 min
T 1/2-SV40-NLS = 37 min
T 1/2-Gtf2h1-NLS = 60 min
ΔT Gtf2h1 to SV40 = 23 min
min post nuclei formation

Supplementary Figure 10: Import kinetics of NLS-GFP fusions correlate with import kinetics of the NLS-origin proteins.
a, We transferred the bioinformatically predicted NLS sequence from proteins assayed in Figure 5, Yy1 (a fast-imported protein $\Delta T_{\text{droplet1/2}}$ = -15.0 min) and the slow-imported proteins Gtf2h1 ($\Delta T_{\text{droplet1/2}}$ = 41.1 min), and Gtf3a ($\Delta T_{\text{droplet1/2}}$ = 33.6 min), to bacterially expressed GFP[19].
b, Experimental procedure. We expressed GFP with the predicted NLS signals from Yy1, Gtf2h1, and Gtf3a to perform nuclear import assays. We added these expressed constructs at the same concentration as the reference SV40-NLS-mCherry into *Xenopus* egg extracts doped with sperm DNA. We collected samples every 5 minutes and imaged nuclear import with confocal microscopy.
c, We monitored the nuclear import kinetic of NLS-GFP and SV40-NLS-mCherry, and fit the data with a sigmoid to extract the time ($T_{1/2}$). We calculate the import time difference (ΔT) between SV40-NLS-mCherry and the NLS of interest to overcome extract variability. Markers represent the raw measurements ($n \geq 5$ replicates), and the box plot shows the spread of measurement data for all the nuclei at each time point. Lines are sigmoid fits. Source and analyzed data are provided as Supplementary Data 7.
d, A scatter plot summarizes the result from the import kinetics of GFP with transferred NLS and import kinetics of the corresponding protein. We observe a good correlation (Pearson correlation of 0.94, two-tailed p-value = 0.006) between $T_{1/2}$ of NLS-GFP constructs measured in the bulk extract and the $\Delta T_{\text{droplet1/2}}$ of proteins measured in the droplet assay. Box plots show the range from the first quartile to the third quartile with the center mark representing the median value. The whiskers represent 1.5*IQR of the distribution.

Supplementary Information Guide

Supplementary Data 1. Results of quantitative proteomic measurement of the relative protein abundance over a developmental time series from the mature oocyte past the ZGA.

Supplementary Data 2. Proteomics quantification of the half-times that proteins enter embryonic nuclei ($T_{\mathrm{embryo1/2}}$) and nuclear fraction (NF) over a developmental time series.

Supplementary Data 3. Proteomic estimation of importin α/β affinity, DNA affinity, and importin α/β+ DNA affinity in arbitrary units.

Supplementary Data 4. Absolute abundance of proteins in the frog eggs reanalyzed with *X. laevis* protein Fasta file based on genome version 9.2[20].

Supplementary movie 1. The movie shows the formation of ~50 µm diameter cell-free droplets of *Xenopus* egg extract in a continuous oil phase using a T-junction microfluidic device.

References

1 Rasar, M. A. & Hammes, S. R. The physiology of the Xenopus laevis ovary. *Methods Mol Biol* **322**, 17-30 (2006). https://doi.org:10.1007/978-1-59745-000-3_2

2 Dumont, J. N. Oogenesis in Xenopus laevis (Daudin). I. Stages of oocyte development in laboratory maintained animals. *J Morphol* **136**, 153-179 (1972). https://doi.org:10.1002/jmor.1051360203

3 Charney, R. M., Paraiso, K. D., Blitz, I. L. & Cho, K. W. Y. A gene regulatory program controlling early Xenopus mesendoderm formation: Network conservation and motifs. *Semin Cell Dev Biol* **66**, 12-24 (2017). https://doi.org:10.1016/j.semcdb.2017.03.003

4 Wuhr, M. *et al.* The Nuclear Proteome of a Vertebrate. *Curr Biol* **25**, 2663-2671 (2015). https://doi.org:10.1016/j.cub.2015.08.047

5 Sasaki, T. & Gilbert, D. M. The many faces of the origin recognition complex. *Curr Opin Cell Biol* **19**, 337-343 (2007). https://doi.org:10.1016/j.ceb.2007.04.007

6 Dutta, A. & Bell, S. P. Initiation of DNA replication in eukaryotic cells. *Annu Rev Cell Dev Biol* **13**, 293-332 (1997). https://doi.org:10.1146/annurev.cellbio.13.1.293

7 Gillespie, P. J., Li, A. & Blow, J. J. Reconstitution of licensed replication origins on Xenopus sperm nuclei using purified proteins. *BMC Biochem* **2**, 15 (2001). https://doi.org:10.1186/1471-2091-2-15

8 Hampoelz, B. *et al.* Pre-assembled Nuclear Pores Insert into the Nuclear Envelope during Early Development. *Cell* **166**, 664-678 (2016). https://doi.org:10.1016/j.cell.2016.06.015

9 Griffin, J. N. *et al.* RAPGEF5 Regulates Nuclear Translocation of beta-Catenin. *Dev Cell* **44**, 248-260 e244 (2018). https://doi.org:10.1016/j.devcel.2017.12.001

10 MacDonald, B. T., Tamai, K. & He, X. Wnt/beta-catenin signaling: components, mechanisms, and diseases. *Dev Cell* **17**, 9-26 (2009). https://doi.org:10.1016/j.devcel.2009.06.016

11 Mathieson, T. *et al.* Systematic analysis of protein turnover in primary cells. *Nat Commun* **9**, 689 (2018). https://doi.org:10.1038/s41467-018-03106-1

12 Newport, J. & Kirschner, M. A major developmental transition in early Xenopus embryos: I. characterization and timing of cellular changes at the midblastula stage. *Cell* **30**, 675-686 (1982). https://doi.org:10.1016/0092-8674(82)90272-0

13 Bilenko, N. Y. & Gallant, J. L. Pyrcca: Regularized Kernel Canonical Correlation Analysis in Python and Its Applications to Neuroimaging. *Front Neuroinform* **10**, 49 (2016). https://doi.org:10.3389/fninf.2016.00049

14 Stuven, T., Hartmann, E. & Gorlich, D. Exportin 6: a novel nuclear export receptor that is specific for profilin.actin complexes. *EMBO J* **22**, 5928-5940 (2003). https://doi.org:10.1093/emboj/cdg565

15 Bohnsack, M. T., Stuven, T., Kuhn, C., Cordes, V. C. & Gorlich, D. A selective block of nuclear actin export stabilizes the giant nuclei of Xenopus oocytes. *Nat Cell Biol* **8**, 257-263 (2006). https://doi.org:10.1038/ncb1357

16 Hodel, M. R., Corbett, A. H. & Hodel, A. E. Dissection of a nuclear localization signal. *J Biol Chem* **276**, 1317-1325 (2001). https://doi.org:10.1074/jbc.M008522200

17 Wilbur, J. D. & Heald, R. Mitotic spindle scaling during Xenopus development by kif2a and importin alpha. *Elife* **2**, e00290 (2013). https://doi.org:10.7554/eLife.00290

18 Brownlee, C. & Heald, R. Importin alpha Partitioning to the Plasma Membrane Regulates Intracellular Scaling. *Cell* **176**, 805-815 e808 (2019). https://doi.org:10.1016/j.cell.2018.12.001

19 Nguyen Ba, A. N., Pogoutse, A., Provart, N. & Moses, A. M. NLStradamus: a simple Hidden Markov Model for nuclear localization signal prediction. *BMC Bioinformatics* **10**, 202 (2009). https://doi.org:10.1186/1471-2105-10-202

20 Wuhr, M. *et al.* Deep proteomics of the Xenopus laevis egg using an mRNA-derived reference database. *Curr Biol* **24**, 1467-1475 (2014). https://doi.org:10.1016/j.cub.2014.05.044
