# Supplementary material for: Differential nuclear import sets the timing of protein access to the embryonic genome
Source: Nat Commun. 2022 Oct 6;13:5887. doi: 10.1038/s41467-022-33429-z (PMC9537182; doi:10.1038/s41467-022-33429-z)
Supplement: Supplementary file 3 — Description of Additional Supplementary Files [file 41467_2022_33429_MOESM3_ESM.pdf]

Description of Additional Supplementary Files

Supplementary Data 1. Results of quantitative proteomic measurement of the relative protein abundance over a developmental time series from the mature oocyte past the ZGA.

Supplementary Data 2. Proteomics quantification of the half-times that proteins enter embryonic nuclei (Tembryo1/2) and nuclear fraction (NF) over a developmental time series.

Supplementary Data 3. Proteomic estimation of importin ŧ/ŧŧ affinity, DNA affinity, and importin ŧ/ŧŧ +DNA affinity in arbitrary units.

Supplementary Data 4. Absolute abundance of proteins in the frog eggs reanalyzed with X. laevis protein Fasta file based on genome version 9.220.

Supplementary movie 1. The movie shows the formation of ~50µm diameter cell-free droplets of Xenopus egg extract in a continuous oil phase using a T-junction microfluidic device.
